# Reassessment strategy to reduce chest imaging in patients with suspected pulmonary embolism and elevated D-dimer: an exploratory cohort study

**DOI:** 10.1016/j.rpth.2026.106821

**Published:** 2026-07-17

**Authors:** Coline Bittermann, Elisa Gicquel, Delphine Douillet, Catherine Ternisien, Quentin Le Bastard, Suzanne Nave, Mathieu Servent, Eric Batard, François Javaudin

**Affiliations:** 1 Nantes Université, CHU Nantes, INSERM, Service des Urgences, CIC 1413, Immuno-infectiologie, Nantes, France; 2 Angers University Hospital, Emergency Department, Angers, France; 3 UMR MitoVasc CNRS 6015—INSERM 1083, Equipe CARME, UNIV Angers, Angers, France; 4 F-CRIN INNOVTE Network, Saint-Etienne, France; 5 Nantes Université, CHU Nantes, Centre de Ressources et de Compétences des Maladies Hémorragiques Constitutionnelles, Nantes, France; 6 Nantes Université, CHU Nantes, Cibles et médicaments des infections et de l’immunité, IICiMed, UR1155, Nantes, France

**Keywords:** chest imaging, clinical decision rules, D-dimer, emergency department, pulmonary embolism

## Abstract

**Background:**

Patients referred by community physicians to the emergency department (ED) for suspected pulmonary embolism (PE) with an elevated D-dimer result often undergo chest imaging, although the appropriate diagnostic strategy in this setting remains unclear.] as per style before objectives

**Objectives:**

This study evaluated whether a standardized 3-step reassessment strategy could reduce chest imaging in these patients.

**Methods:**

Using data from an observational cohort study conducted in 2 French EDs between May 2022 and May 2023, a standardized 3-step reassessment strategy was retrospectively applied. The strategy combined (1) clinical probability reassessment, (2) adjustment of community laboratory D-dimer thresholds, and (3) repeat D-dimer testing in the ED using the VIDAS assay when indicated. Adult patients referred for suspected PE with a community D-dimer level of >500 ng/mL were included. The primary outcome was the proportion of chest imaging studies that would have been avoided.

**Results:**

Seventy-five patients were analyzed (median age, 59 years; 57% women). Application of the reassessment strategy would have avoided chest imaging in 37 patients, corresponding to an absolute reduction of 49 percentage points (95% CI, 37-60). Imaging avoidance occurred at step 1 in 11 patients (absolute reduction, 15 percentage points; 95% CI, 7-24), at step 2 in 14 patients (19 percentage points; 95% CI, 10-29), and at step 3 in 12 patients (16 percentage points; 95% CI, 8-26). The failure rate was 1% (95% CI, 0-7).

**Conclusion:**

In patients referred to the ED for suspected PE with an elevated community D-dimer result, application of a standardized reassessment strategy could reduce chest imaging by approximately half. The safety and generalizability of this approach warrant evaluation in larger, prospectively designed studies.

## Essentials

• D-dimer assays are not analytically equivalent, and their results are not interchangeable.
• We developed a 3-step strategy for suspected pulmonary embolism, combining clinical probability assessment, adjusted D-dimer thresholds, and selective repeat testing using the VIDAS assay.
• Retrospective application of this strategy in the emergency department would have avoided approximately half of chest imaging examinations.
• The safety of this strategy requires evaluation in larger prospective studies.

## Graphical abstract

**Figure:**
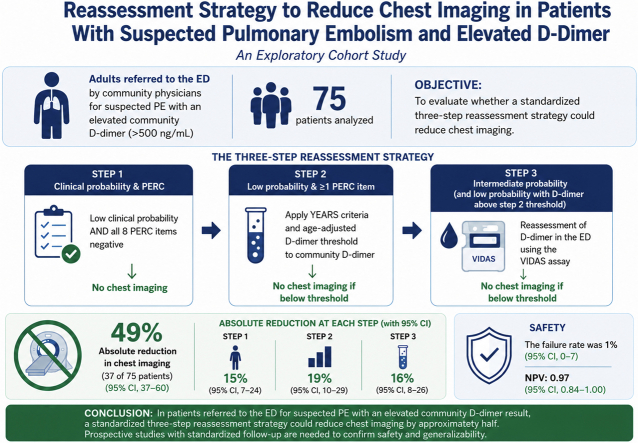

## 1 Introduction

Evaluation of patients with suspected pulmonary embolism (PE) relies on a structured, stepwise diagnostic strategy [[1], [2], [3]]. The initial step consists of assessing the clinical pretest probability, categorized as low, intermediate, or high, using validated clinical prediction rules such as the Wells score or the revised Geneva score, or, more commonly in clinical practice, physician gestalt. In patients with a low pretest probability (<15%) who meet all 8 negative criteria of the Pulmonary Embolism Rule-out Criteria (PERC) [4], PE can be safely excluded without further diagnostic testing [3,5]. Except for patients with a high pretest probability, remaining patients undergo D-dimer testing, with diagnostic thresholds adapted according to clinical probability, the YEARS algorithm, or patient age [3,[6], [7], [8]]. On the basis of the D-dimer result, chest imaging, either computed tomographic pulmonary angiography (CTPA) or ventilation–perfusion (V/Q) scintigraphy, is indicated or can be safely avoided.

This comprehensive diagnostic approach, from initial clinical assessment to definitive testing, is routinely applied in the emergency department (ED). However, a substantial proportion of patients are referred to the ED by general practitioners primarily for chest imaging after an initial outpatient evaluation for suspected PE and the identification of an elevated D-dimer level in a community laboratory. In this context, when patients arrive in the ED with a D-dimer result already available from outside the hospital, it remains unclear whether emergency physicians should restart the diagnostic workup from the beginning or proceed directly to chest imaging.

In addition, clinically relevant interassay variability exists among D-dimer assays, which rely on heterogeneous analytical methodologies [9,10]. Importantly, D-dimer results are not interchangeable between assays [9,11], and some methods exhibit greater variability, potentially leading to patient misclassification and missed diagnoses of PE [12]. The bioMérieux Vitek Immunodiagnostic Assay System (VIDAS) assay is an enzyme-linked fluorescent immunoassay (ELFA) with diagnostic sensitivity at least comparable with that of microplate ELISA, historically considered the reference standard [11,13]. Some clinical guidelines vary in their recommendations regarding the use of specific D-dimer assays according to pretest probability, with some restricting assay types and others making no such distinction [3,[14], [15], [16]]. The aim of this study was to assess the reduction of chest imaging using a standardized reassessment strategy for ambulatory patients referred for chest imaging after an elevated D-dimer from community laboratories using immunoturbidimetric assays.

## 2 Methods

### 2.1 Design

This observational cohort study was conducted between May 2022 and May 2023 in the ED of 2 university-affiliated tertiary care hospitals in France. All patients were evaluated by attending emergency physicians according to routine clinical practice. During the study period, relevant clinical data, including physician gestalt assessment of pretest probability, PERC, and YEARS criteria, were prospectively collected for all patients referred for suspected PE with an elevated D-dimer result (>500 ng/mL) obtained in a community laboratory using immunoturbidimetric assays. For each patient, D-dimer levels were systematically remeasured in the hospital laboratory using the bioMérieux VIDAS assay.

The standardized reassessment strategy was applied retrospectively to this cohort. Importantly, the diagnostic approach used in routine clinical practice was not dictated by the study protocol. Emergency physicians remained free to manage patients according to usual care, including decisions regarding chest imaging.

### 2.2 Population

Adult patients (≥18 years) referred to the ED by outpatient community physicians for suspected PE after an elevated D-dimer result (>500 ng/mL) obtained in a community laboratory using immunoturbidimetric assays were included. Exclusion criteria were a high clinical gestalt probability of PE determined by the emergency physician (patients excluded at presentation and therefore not counted in the cohort), ongoing anticoagulant therapy for >24 hours, and pregnancy.

### 2.3 Reassessment strategy

The first step consisted of an emergency physician’s assessment of clinical gestalt probability and evaluation of the PERC score. If the clinical gestalt probability was low and all 8 PERC items were negative, chest imaging was considered unnecessary.

The second step applied to patients with a low gestalt probability of PE but at least 1 positive PERC item. In these patients, the community D-dimer threshold was defined according to the YEARS criteria or patient age. If no YEARS items were present, the threshold was set at 1000 ng/mL. If 1 or more YEARS items were present, the D-dimer threshold was age adjusted: 500 ng/mL for patients aged ≤50 years and age × 10 ng/mL for those aged >50 years. Patients whose community D-dimer result was below the relevant threshold were considered not to require chest imaging. Patients whose community D-dimer value exceeded the defined threshold proceeded to step 3.

**Figure 1:**
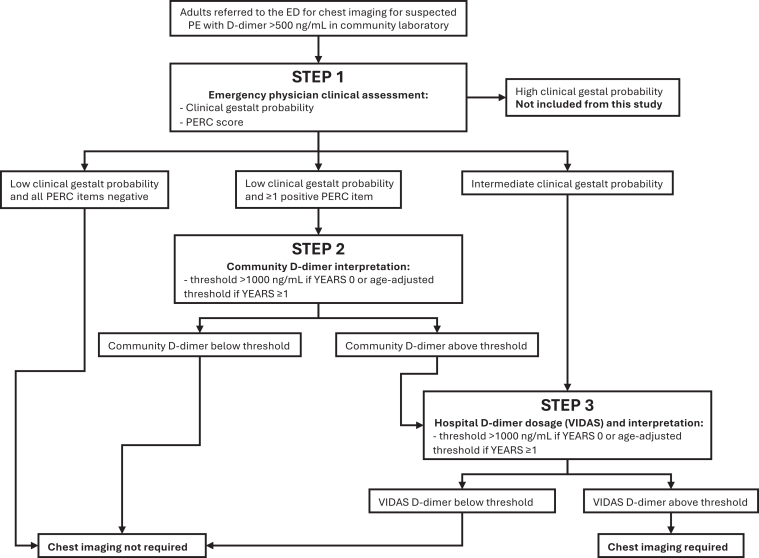
Standardized 3-step reassessment strategy for patients referred to the emergency department (ED) for chest imaging after an elevated community D-dimer result and suspected pulmonary embolism. PERC, Pulmonary Embolism Rule-out Criteria; VIDAS, Vitek Immunodiagnostic Assay System.

The third step concerned patients with an intermediate clinical gestalt probability and those with a low probability but a community D-dimer value above the threshold defined in step 2. In these patients, a repeat D-dimer measurement was performed in the hospital laboratory using the VIDAS assay. The same thresholds as in step 2 were then applied. If the VIDAS D-dimer result was below the threshold, these patients were considered not to require chest imaging. An overview of the reassessment strategy is presented in Figure 1.

### 2.4 Outcomes

The primary outcome was the proportion of chest imaging studies (CTPA or V/Q scintigraphy) that would have been avoided by application of the 3-step reassessment strategy. For descriptive purposes, we also quantified the number and percentage of imaging studies that would have been avoided at each individual step of the strategy. Secondary outcomes included the safety of the reassessment strategy, assessed by comparing the strategy-based imaging classification with the final ED diagnosis, as well as agreement between community laboratory and VIDAS D-dimer measurements.

### 2.5 Ethics

In accordance with French regulations for observational studies, patients received written information indicating that data from their medical charts would be used for research. Ethical approval was obtained from the Nantes University Hospital Institutional Review Board (No. 22-03-911).

### 2.6 Statistical analysis

The primary outcome was evaluated by calculating the absolute difference in percentage points between the proportion of chest imaging avoided with the reassessment strategy and the proportion that would have occurred with systematic chest imaging, which was the reason for referral to the ED. Further, 95% CI for these differences were calculated using the exact method. To detect a clinically important reduction in chest imaging of >5 percentage points at each of the 3 steps, the required sample size was calculated to be 80 patients. The secondary outcome was assessed by estimating sensitivity, specificity, positive predictive value, and negative predictive value for PE. Patient characteristics were described as number (percentage) for categorical variables and median (IQR) for quantitative variables. A Bland–Altman analysis compared VIDAS D-dimer levels with community D-dimer levels. The mean bias (SD) and 95% CI for the limits of agreement were reported. All tests were 2-sided, and a *P* value of <.05 was considered statistically significant. Analyses were performed with Stata software, version 16.0 (StataCorp), and R software, version 4.5.0 (R Foundation for Statistical Computing).

## 3 Results

A total of 76 patients were included during the study period. One patient was excluded because of ongoing anticoagulant therapy for >24 hours, leaving 75 patients for analysis. The median age was 59 years (IQR, 38-74 years), and 57% were women. The median community D-dimer level was 1181 ng/mL (IQR, 719-2360 ng/mL). Most patients (68% [*n* = 51]) had a low clinical gestalt probability of PE, whereas approximately one-third (32% [*n* = 24]) had an intermediate probability.

### 3.1 Primary outcome

**Figure 2:**
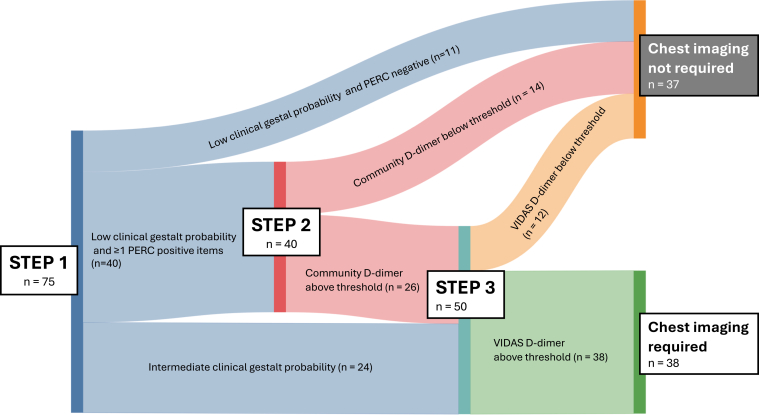
Patient flow across the standardized 3-step reassessment strategy. Branch widths in the Sankey diagram are proportional to the number of patients in each pathway. PERC, Pulmonary Embolism Rule-out Criteria; VIDAS, Vitek Immunodiagnostic Assay System.

Application of the reassessment strategy would have avoided chest imaging in 37 of 75 patients (Figure 2), corresponding to an absolute reduction of 49 percentage points (95% CI, 37-60) compared with the systematic chest imaging that would have been performed based on the initial referral indication. Patients who did not require chest imaging were significantly younger (median age, 50 vs 69 years) and had lower community-based D-dimer levels (median, 750 vs 1926 ng/mL) (Table 1). Among these patients, 11 imaging studies would have been avoided at step 1 (absolute reduction, 15 percentage points; 95% CI, 7-24), 14 at step 2 (19 percentage points; 95% CI, 10-29), and 12 at step 3 (16 percentage points; 95% CI, 8-26).

**Table 1:** Patient characteristics, clinical presentation, and ED outcomes according to whether chest imaging was required under the reassessment strategy.

Characteristic	Chest imaging not required according to the reassessment strategy (*n* = 37)	Chest imaging required according to the reassessment strategy (*n* = 38)
Patient characteristics and history		
Age (y)	50 (33-68)	69 (42-80)
Female sex	20 (54)	23 (61)
Chronic respiratory insufficiency	2 (5)	4 (11)
Chronic or ischemic heart disease	2 (5)	8 (21)
Stroke	0 (0)	0 (0)
Chronic kidney failure	1 (3)	7 (18)
Past PE and/or DVT	1 (3)	6 (16)
Cancer	0 (0)	3 (8)
Immobilization or surgery < 1 mo	1 (3)	1 (3)
Exogenous estrogen use	3 (8)	3 (8)
Clinical presentation		
Chest pain	26 (70)	18 (47)
Dyspnea	17 (46)	29 (76)
Syncope	2 (5)	2 (5)
Hemoptysis	0 (0)	1 (3)
Heart rate (bpm)	83 (71-88)	84 (72-91)
Respiratory rate (cycles/min)		
Systolic blood pressure (mm Hg)	136 (123-155)	136 (120-155)
Oxygen saturation (%)	97 (97-99)	97 (95-98)
Clinical signs of DVT	0 (0)	2 (5)
Temperature ≥ 38 °C within the previous 24 h	2 (5)	1 (3)
Community D-dimer level (ng/mL)	750 (618-1181)	1926 (1100-3000)
VIDAS D-dimer level (ng/mL)	603 (452-779)	2207 (1439-2725)
ED final diagnosis		
Pulmonary embolism	1 (3)	13 (34)
Pneumonia	1 (3)	3 (8)
Pleurisy	1 (3)	2 (5)
Chest wall pain	4 (11)	2 (5)
Nonspecific chest pain	11 (30)	1 (3)
Vasovagal syncope	1 (3)	0 (0)
Anxiety	3 (8)	1 (3)
Bronchitis	3 (8)	6 (16)
Other	12 (32)	10 (26)
ED discharge		
Hospital admission	3 (8)	14 (37)
Discharged home	34 (92)	24 (63)

Values are given as n (%) or median (IQR).

DVT, deep vein thrombosis; ED, emergency department; PE, pulmonary embolism.

### 3.2 Secondary outcomes

In this cohort, and independently of the retrospective reassessment strategy, 52 patients underwent CTPA and 1 underwent V/Q scintigraphy, whereas 22 patients did not undergo chest imaging. Fourteen cases of PE were diagnosed in the ED. Among patients who would not have required chest imaging according to the reassessment strategy (*n* = 37), 1 case of PE was identified, corresponding to a failure rate of 1% (95% CI, 0-7). This patient was a 69-year-old woman with an intermediate clinical gestalt probability. Her community-based D-dimer level was 660 ng/mL, and the repeat VIDAS D-dimer level was 897 ng/mL; the diagnostic threshold had been set at 1000 ng/mL because no YEARS criteria were present. The PE was classified as low risk (simplified Pulmonary Embolism Severity Index score, 0) and was managed on an outpatient basis. Among the patients for whom chest imaging was required according to the reassessment strategy (*n* = 38), 13 cases of PE (34%) were diagnosed. The sensitivity of the reassessment strategy for the diagnosis of PE was 0.93 (95% CI, 0.66-1.00), the specificity 0.59 (95% CI, 0.46-0.71), the positive predictive value 0.34 (95% CI, 0.27-0.42), and the negative predictive value 0.97 (95% CI, 0.84-1.00) (Table 2).

**Table 2:** Contingency table comparing chest imaging requirement according to the reassessment strategy with the final emergency department diagnosis of pulmonary embolism.

Chest imaging requirement according to the reassessment strategy	PE diagnosis in the ED	No PE diagnosis in the ED
Chest imaging required	13	25
Chest imaging not required	1	36

ED, emergency department.

The median community D-dimer level was 1181 ng/mL (IQR, 719-2360 ng/mL) compared with 1006 ng/mL (IQR, 603-2274) for the VIDAS D-dimer; the corresponding means were 1945 ± 2402 ng/mL vs 1529 ± 1383 ng/mL, respectively. Using the VIDAS assay as the reference, the mean bias was −416.3 ± 2252 ng/mL, with 95% limits of agreement of −4831 to 3998 ng/mL. Notably, 1 marked outlier was observed, with a community D-dimer level of 18,600 ng/mL compared with a VIDAS D-dimer level of 578 ng/mL.

## 4 Discussion

In this study of patients referred to the ED for chest imaging because of suspected PE and an elevated community D-dimer result, the retrospective application of a standardized reassessment strategy, based on clinical assessments and data prospectively collected by attending emergency physicians, would have reduced the number of chest imaging examinations by approximately half. This 3-step strategy combined structured clinical reassessment, adjustment of the D-dimer threshold, and, when indicated, repeat D-dimer testing in the ED using the VIDAS assay. Each component of the strategy contributed to a significant reduction in imaging requirements.

The first step was consistent with the approach evaluated in the PROPER trial, which enrolled patients with a low clinical gestalt probability of PE and demonstrated that application of the PERC rule safely avoided ∼10% of chest imaging examinations [5]. In the PERCEPIC study, 32% of ED patients with a low implicit clinical probability of PE were PERC negative before D-dimer testing [17]. These trials included all ED patients with suspected PE, whereas our study focused specifically on patients initially evaluated by a community physician who had a D-dimer level of >500 ng/mL before referral to the ED. Despite this difference in population, likely associated with a higher pretest probability, step 1 of the reassessment strategy still avoided ∼15% of chest imaging.

The second step applied the YEARS rule combined with an age-adjusted D-dimer threshold, following the design of the MODIGLIANI study [6]. That study reported a 9% reduction in chest imaging among patients with a positive PERC rule and a low or intermediate clinical gestalt probability of PE. In the YEARS study, in which the D-dimer threshold was not adjusted for age when at least 1 YEARS item was present, the reduction in CTPA use was estimated at 14% [18]. In our cohort, adjusting the community-based D-dimer threshold at step 2 among patients with low clinical gestalt probability yielded a 19% reduction in chest imaging. However, the MODIGLIANI trial did not account for assay-specific differences in D-dimer performance, although not all methods are equivalent at the diagnostic thresholds used to exclude venous thromboembolism [9]. A secondary analysis of the ADJUST-PE study, which used an age-adjusted cutoff (age × 10 ng/mL for patients older than 50 years), showed that assays such as STA-Liatest or Tina-quant occasionally produced false-negative results, whereas other methods, including VIDAS, did not [12]. Because the VIDAS assay is regarded as one of the most sensitive D-dimer tests [14,16], we selected it as the reference method for patients with an intermediate clinical gestalt probability or for those with community D-dimer levels above the step 2 adjusted thresholds. This choice was intended to maximize the safety of our strategy while reducing the need for chest imaging. The only PE diagnosed in the ED that would not have required chest imaging according to the reassessment strategy was a PE without severity criteria, managed on an outpatient basis. This represented a failure of the YEARS score, with no positive items reported by the emergency physician. In such patients, the community laboratory D-dimer level was below the age-adjusted cutoff (age × 10), whereas the VIDAS D-dimer level was above this threshold but remained below 1000 ng/mL. Similar concerns have previously been raised regarding patients with no YEARS items and a D-dimer level below 1000 ng/mL but above the age-adjusted cutoff, a subgroup in which caution has been recommended despite the overall safety of the YEARS strategy [19]. Although some immunoturbidimetric assays have shown performance comparable with ELFA methods such as VIDAS in certain studies [13,20], the VIDAS method remains widely regarded as a reference standard. Based on data from >700 laboratories, the bioMérieux VIDAS assay has demonstrated the highest analytical precision at the diagnostic threshold for venous thromboembolism [9]. Several guidelines specifically recommend ELFA assays and advise greater caution with immunoturbidimetric methods, which are considered less reliable for safely ruling out PE, particularly in patients with intermediate clinical probability or when an age-adjusted D-dimer threshold is applied [14,16].

A meta-analysis reported that the YEARS strategy had a failure rate of 0.25% (95% CI, 0.07-0.94) in primary care settings. However, among patients referred by general practitioners based on a clear clinical suspicion of PE, the failure rate increased to 2.10% (95% CI, 1.59-2.75) [21]. In the MODIGLIANI study, the combination of the YEARS strategy with an age-adjusted D-dimer threshold yielded a failure rate of 0.15% (95% CI, 0.00-0.86) among patients presenting to the ED (self-referred or referred) with symptoms suggestive of PE, including new-onset or worsening shortness of breath, chest pain, or syncope [6]. Another recommended strategy is the PEGeD algorithm, which adapts the D-dimer threshold according to the Wells score [22,23]. However, its failure rate was not lower than that of the YEARS strategy, in either the primary care or the secondary care (0.43% and 3.06%, respectively) [21]. Finally, the third step of our strategy, which involved reassessment of D-dimer levels in the ED using the VIDAS assay, contributed to a further 16% reduction in chest imaging.

### 4.1 Limitations

This study has several limitations. First, the reassessment strategy was applied retrospectively to the entire cohort, assuming full adherence by emergency physicians to the 3-step approach when determining the need for chest imaging. This post hoc application may overestimate the proportion of imaging that could be avoided, because real-world implementation is likely to involve incomplete compliance, variability in clinical decision making, and practical workflow constraints.

Second, all repeat D-dimer testing in the ED was performed using the VIDAS assay, which is recognized for its high analytical sensitivity and precision. The performance of the third step of the reassessment strategy may differ with other D-dimer assay methods, limiting the generalizability of our findings.

Third, assessment of the safety of the reassessment strategy was limited because PE was defined solely by the final ED diagnosis at discharge, without systematic follow-up. Patient management, including diagnostic testing, was left at the discretion of the treating emergency physician. As a result, delayed diagnoses or missed thromboembolic events after ED discharge may not have been captured. In addition, the small sample size and limited number of PE events precluded robust evaluation of the safety of the reassessment strategy. Therefore, safety outcomes should be considered exploratory only and require confirmation in larger prospective studies with standardized follow-up.

Finally, emergency physicians were not blinded to the community D-dimer result, which may have introduced incorporation bias in the assessment of clinical gestalt probability.

## 5 Conclusion

The retrospective application of a 3-step reassessment strategy would have led to a substantial reduction in chest imaging among patients referred to the ED by community physicians for suspected PE with an elevated community laboratory D-dimer result. However, the limited sample size and retrospective design preclude definitive conclusions regarding the safety of this approach. Larger prospective studies with standardized follow-up are required before implementation in clinical practice.
